# Editorial: Subcortical and spinal control of motor networks across vertebrates

**DOI:** 10.3389/fnana.2024.1378811

**Published:** 2024-02-21

**Authors:** Jean-Luc Boulland, Marie-Claude Perreault

**Affiliations:** ^1^Division of Physiology, Department of Molecular Medicine, Institute of Basic Medical Sciences, University of Oslo, Oslo, Norway; ^2^Department for Immunology, Clinic for Laboratory Medicine, Oslo University Hospital, Oslo, Norway; ^3^Department of Cell Biology, Emory University School of Medicine, Atlanta, GA, United States

**Keywords:** brainstem, spinal cord, network interactions, essential movements, gazing, breathing, locomotion

Essential motor functions, such as feeding, breathing, and locomotion, are produced by networks of neurons located in the brainstem and spinal cord. Understanding the function of these circuits requires studying their cellular organization and connectivity. Brainstem and spinal cord motor networks are often studied as separate or standalone entities. A typical example is the brainstem network that processes retinal image stabilization, which involves converting motion-related sensory feedback into ocular motor commands. However, from a broader physiological perspective, these networks are not isolated; they interact with each other. The present Research Topic highlights recent advances in our understanding of subcortical and spinal circuits producing essential motor functions and provides new insights on how these motor networks interact and coordinate during movement. Twenty-nine authors contributed to this Research Topic with three original research articles and three reviews. Their contributions are summarized below.

## Development and makeup of spinal motor networks

Spinal networks are essential for a plethora of motor and autonomic functions. With the support of early developing descending inputs, these networks produce basic motor activities already from birth. During postnatal development, they are supplemented by sensory feedback, leading to more robust and adapted movements. Hence, basic motor activities transition into controlled movements. Laliberte et al. use chemogenetics, immunohistochemistry, synaptic quantification, and reflex testing to investigate the mechanisms that underlie the developmental transition of palmar grasp reflex—flexion of digits in response to palmar skin touch—into smooth and coordinated grasping movement. The study associates increased presynaptic inhibition of sensory inputs to dorsal horn dI3 interneurons in the cervical cord and maturation of grasping, supporting the idea that postnatal maturation of motor control is driven by changes in sensorimotor integration.

Dl3 interneurons are merely one cardinal group of spinal interneurons among many (Wilson and Sweeney, 2023). Furthermore, each cardinal group is far from homogenous and likely contains interneuron subgroups that may be associated with different motor functions. Such diversity represents a key challenge for our understanding of the spinal control of movement in both newborns and adults. Garcia-Ramirez et al. investigate Shox2 (V2d) interneurons in the lumbar cord, a population of excitatory interneurons with ipsilateral projections to motoneurons that plays a variety of roles during locomotion. However, Shox2 population partly overlaps with Chox10 (V2a) population. To distinguish the Shox2 from the Chx10 population, the authors initially used a method that compared the firing response of adult Shox2 and Chx10 neurons to suprathreshold depolarizing current steps. This comparison revealed four firing response types (tonic, initial doublet, initial burst, and delayed firing) but was unsatisfactory as each firing type was present in both groups. They then conducted unbiased cluster analysis on the same populations considering a set of 12 passive and active membrane properties. Using this approach, they effectively identified clusters composed exclusively of Shox2 neurons (Shox2+ Chx10–). Based on these findings, the authors argue that computational clustering based on electrophysiological variables may powerfully complement classification of spinal interneurons by molecular and genetic markers.

## Interactions between motor networks

The Mesencephalic Locomotor Region (MLR), comprising the cuneiform and pedunculopontine nuclei, can initiate and influence locomotion. However, it has long been known, and perhaps largely forgotten, that the role of the MLR goes well-beyond locomotion, influencing arousal, cardiovascular, and respiratory functions. Noga and Whelan revisit the MLR's diverse functions, discussing its various inputs and projections and its role in coordinating autonomic and motor behaviors. Noga and Whelan conclude this review with a translational perspective in connection with surgical implantation of deep brain stimulation electrodes to address motor disorders.

This work resonates with the review by Juvin et al., who detail the mechanisms by which locomotor and respiratory networks become linked—a phenomenon known as locomotor-respiratory coupling. Besides biomechanics mechanisms that can vary among species, the authors describe several neurogenic mechanisms, some of which involve the MLR.

In turn, Missaghi et al. combine lesion, anatomy, pharmacology, and electrophysiology methods to re-examine the spatial organization and characterize the operation of the brainstem centers responsible for fast and slow respiration in lampreys. The study highlights various levels of sophistication in the control of respiration in vertebrates.

As another example of interactions between motor networks, Straka et al. review the role of efference copies—internal copies of movement-producing signals—in stabilizing the gaze during locomotion at different stages of development. The review highlights that during locomotor activity, efference copies can directly offset visual perturbations and drive compensatory eye adjustments, to stabilize vision during movement. The authors also describe how horizontal vestibulo-ocular reflexes are selectively suppressed during intense locomotor activity by efference copies that reduce mechano-electrical encoding at the vestibular sensory periphery. They suggest that this attenuation enables the system to prioritize the predictive locomotor commands over the reactive sensory feedback, leading to more efficient and coordinated locomotion.

This Research Topic offers significant insights into the intricate network of motor control spanning the subcortical and spinal regions across vertebrates. Through a combination of original research and reviews, the works presented illuminate the complexity and interconnectivity of motor networks responsible for essential functions such as breathing and walking. Key findings include the developmental transitions in spinal motor networks facilitated by sensory feedback, the identification of distinct interneuron populations critical for movement control, and the multifaceted roles of the MLR in coordinating motor and autonomic behaviors. Additionally, the exploration of neurogenic mechanisms underlying locomotor-respiratory coupling and the influence of efference copies on gaze stabilization during locomotion provide a deeper understanding of how vertebrates achieve adaptive movements. This research not only advances our understanding of motor network interactions but also opens avenues for translational approaches to treat motor disorders.

## Author contributions

J-LB: Writing—original draft, Writing—review & editing. M-CP: Writing—original draft, Writing—review & editing.
